# A Review of 105 Subscapular-Based Flaps Harvested Using a New Dorsal Decubitus Position: How Far Can We Go?

**Published:** 2013-02-28

**Authors:** Laurence S. Paek, Olivier Boa, Marc Revol, Jean-Marie Servant, Patrick G. Harris, M. Alain Danino

**Affiliations:** ^a^Hôpital Notre-Dame, Centre Hospitalier de l’Université de Montréal, Division of Plastic Surgery, Université de Montréal, Montreal, Quebec, Canada; ^b^Hôpital Saint-Louis, Division of Plastic and Reconstructive surgery, Université de Paris, Paris, France.

## Abstract

**Objective:** Interest in flaps based on the subscapular vascular system has decreased because of the need for intraoperative patient repositioning and the inability to employ a simultaneous 2-team approach. The aims of this study are to review our experience using dorsal decubitus patient positioning for subscapular-based flap harvest and to demonstrate the effectiveness and safety of this approach. **Methods:** A retrospective review of all subscapular-based flap cases performed by the senior author at 2 hospital centers from 1995 to 2010 was conducted. Variables studied included indications for reconstruction, flap characteristics, and postoperative complications. A longitudinal roll placed between the scapulae as well as an optional perpendicularly placed shoulder roll are used to achieve dorsal decubitus patient positioning. **Results:** One hundred five flaps were performed during the study period, and dorsal decubitus positioning was used in all cases. Eighty-four flaps were free and 21 were pedicled. Indications for reconstruction included cancer resection (n = 58), trauma (n = 32), infection (n = 9), and others (n = 6). A simultaneous 2-team approach was carried out in 70 cases. Major complications included 9 cases of arterial or venous thrombosis/insufficiency, 2 of which resulted in total flap failure. Intraoperative conversion to lateral decubitus positioning was never required. **Conclusions:** Dorsal decubitus harvesting for subscapular-based flaps is a practical and effective technique that enables a simultaneous 2-team approach in complex reconstructive cases. Previous limitations of these highly versatile flaps, such as the need for intraoperative patient repositioning, can thus be avoided. This approach is employed for all subscapular-based flap reconstructions performed by the senior author.

In reconstructive surgery, the subscapular vascular system is an important anatomical crossroad that provides a wide variety of flaps: the latissimus dorsi flap (LDF), scapular or parascapular fasciocutaneous flaps, serratus muscle flap, scapular bone flap, and thoracodorsal artery perforator (TAP) flap. The LDF, harvested in either muscular or musculocutaneous forms, remains the most commonly used flap of the subscapular system.

First described in 1906 by Iginio Tansini to permanently cover deficits of the anterior thorax following radical mastectomies,^1^ the latissimus dorsi (LD) gained popularity in 1976 with Olivari, who utilized it for the reconstruction of irradiated thoracic tissues as a pedicled flap.^2^ The LDF's applications, largely as a result of the evolution of microsurgery, have since broadened; it is now a valuable option for coverage of major tissue deficits in the head and neck^3^^,^^4^ as well as on the chest wall and lower extremities.^5^^,^^6^ The LDF's applications also extend to the coverage and functional restoration of the arm and shoulder.^7^^-^9

The LDF has very predictable vascularization with a robust pedicle measuring up to 8 cm in length^10^ and 4 mm in diameter. It can be used as either a pedicled or free flap providing as much as 30 × 40 cm of muscle, fat, and cutaneous tissue. Despite its many advantages and wide range of applications, the LDF does present certain drawbacks. With the exception of high-performance athletes, harvesting the flap often results in negligible donor site functional deficits due to compensation by the other girdle muscles. However, in one series of 85 female patients who underwent LD muscle transfer, Adams et al report that up to 39% complained of moderate shoulder weakness, 50% experienced some back numbness or tightness, and 22% found their donor site scar to be unacceptable.^11^ Other limitations include the high incidence of postoperative seroma and, importantly, the necessity of a lateral decubitus patient position for harvesting. While newer endoscopic surgical techniques appear to minimize the donor site scar and lower overall complication rates, the harvesting position remains problematic, particularly in conjoint cases when 2 teams are working simultaneously.^12^^-^14

Over the last few decades, interest in flaps based on the subscapular vascular system has decreased. We believe this trend is largely due to inconveniences related to the traditional lateral decubitus harvesting position with which intraoperative position changes are necessary and simultaneous 2-team operations are often impractical. The aims of this study are to review our experience using an alternative dorsal decubitus patient position for raising a wide range of subscapular-based flaps and to demonstrate the effectiveness, safety, and potential limitations of this approach.

## METHODS

A retrospective review of all subscapular-based flap cases performed by the senior author at 2 hospital centers between 1995 and 2010 was conducted. All patient charts were reviewed to determine the underlying etiology and specific location of each reconstructed tissue deficit. Whether a 1- or 2-team approach was used in each case was also documented. Other variables studied included age; sex; mode of tissue transfer; flap composition; and size/quantity of the cutaneous, muscular, and osseous components. All incidences of complications were documented to determine the overall success rate of these procedures. Complications were classified as either minor or major. Major complications were defined as those cases requiring operative intervention. Only patients having undergone a minimum of 1-year postoperative follow-up were included in this study.

### Dorsal decubitus technique

#### Preoperative markings and patient setup

**Figure 1:**
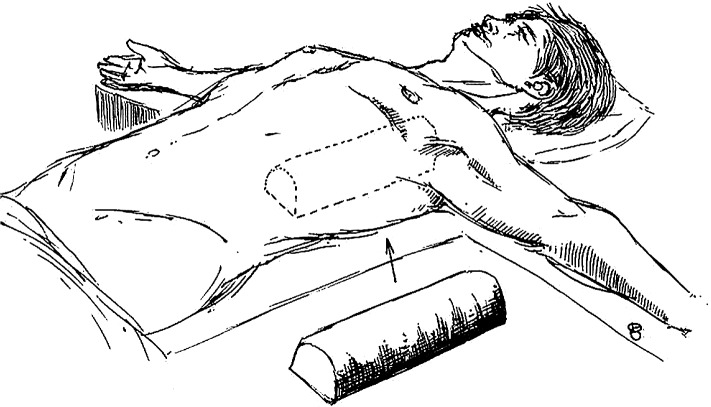
Illustration of a patient in the dorsal decubitus position. One cushion is placed under the back, longitudinally oriented along the spine.

**Figure 2:**
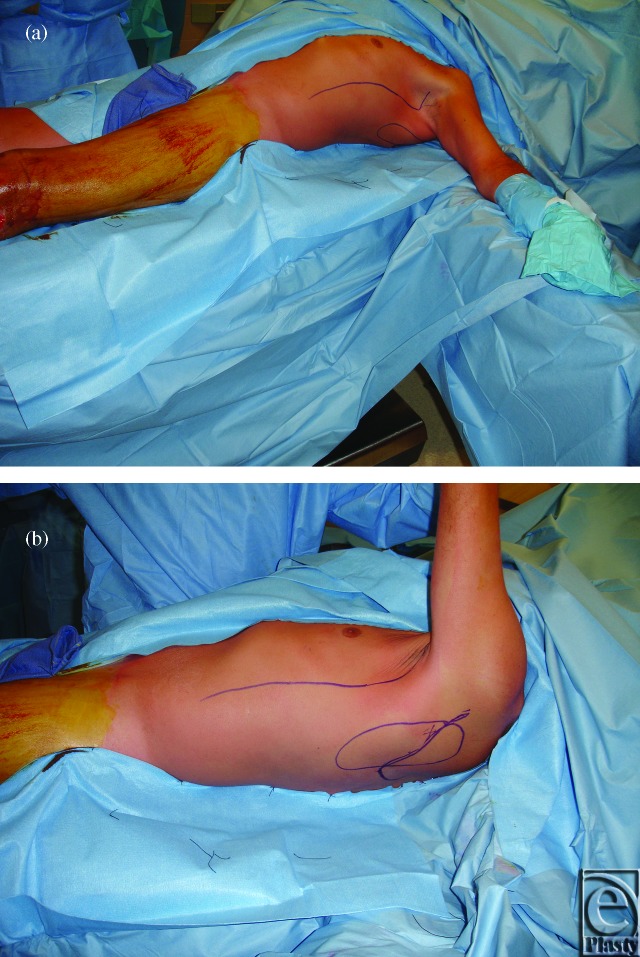
(*a*) Patient is shown installed in the dorsal decubitus position following completion of prepping and sterile drape placement. (*b*) Close-up view of dorsal decubitus position.

With the patient standing, the usual key landmarks are identified: the inferior border of the scapula, the posterior midline corresponding to the spinous processes, and the superior border of the iliac crest. The anterior border of the muscle is then delineated while the patient places his or her hands on the hips and pushes downward toward the pelvis. Once the patient is fully anesthetized, a first cushion is placed under the spine along the longitudinal axis of the body and, if deemed necessary to optimize exposure, an optional second cushion is positioned perpendicularly just under the scapular belt, thereby forming a “T-Roll.” Positioning the patient in this manner permits full access to all components of the subscapular vascular system. Figure 1 demonstrates the proper placement of the longitudinal roll for patient dorsal decubitus positioning. Skin preparation is performed to include the ipsilateral halves of the anterior and posterior thoracic walls as well as the entire ipsilateral upper extremity. The sterile drapes are fixed to the posterior midline of the patient (Fig 2). Prepping the arm is essential because it permits the assistant to mobilize the arm intraoperatively, thereby facilitating pedicle dissection up to the axillary vessels if a longer pedicle is needed. The side of flap harvest is chosen on the basis of the location of the deficit to be reconstructed, whether a simultaneous 2-team approach is envisioned or not, and whether a pedicled or free flap is planned.

#### Operative technique

Prior to incision, the anterior border of the LD muscle is always verified via digital palpation as it can be displaced posteriorly in this position; the anterior border is then injected with a diluted solution of 500 mL of normal saline and 1 mg of epinephrine 1:1000. The initial skin incision is then made according to the composition of the flap to be harvested. If only a muscular component is envisioned, the incision is made just anteriorly to the demarcated border of the LD muscle; otherwise, it is made with respect to the area of the planned skin paddle.

In the case of LD musculocutaneous flaps, subcutaneous dissection is performed until the anterior border of the LD is identified; in cases not requiring a cutaneous component, a direct incision is made to the deep fascia and suprafascial dissection is performed as needed. Once the anterior border of the LD is reached, the areolar plane distinguishing it from the serratus muscle is identified; the LD's natural adhesions to the serratus muscle slips are then separated. At this stage, it is useful to suture the skin of the thoracic wall onto itself; doing so optimizes exposure and allows the assistant to exert countertraction on the serratus fascia as pedicle dissection is carried out into the axillary region in the plane between the serratus anterior muscle and the LD. The first vascular branch encountered is the serratus branch and it is normally ligated, unless a serratus muscle component is planned.

**Figure 3:**
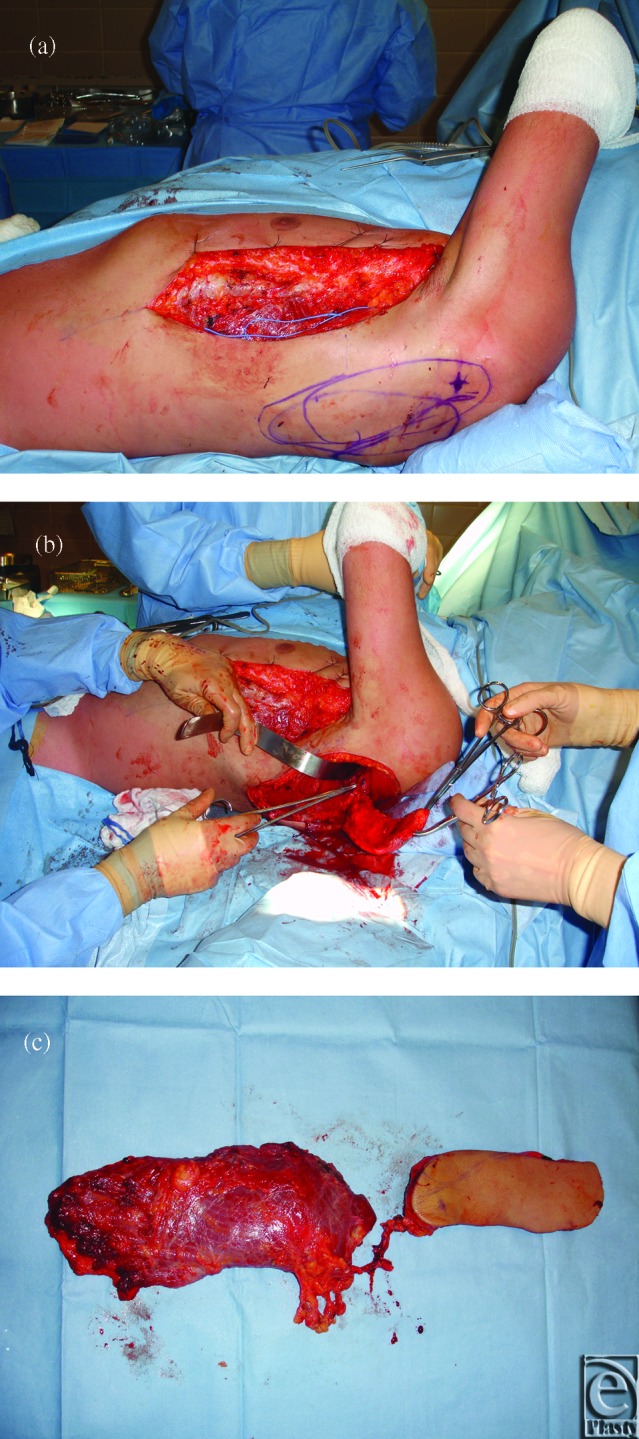
Chimeric subscapular-based flap harvesting in the dorsal decubitus position. (*a*) Harvesting of the latissimus dorsi musculocutaneous flap component. (*b*) Harvesting of the fasciocutaneous parascapular flap component. (*c*) Final chimeric flap after pedicle transection prior to inset. The subscapular artery pedicle was dissected up to the axillary junction.

In cases where a chimeric osseous flap is planned, care is taken to preserve the angular branch; when present, the angular artery may originate from either the thoracodorsal artery or the serratus branch. Once the branches of the subscapular system are identified, the minor perforating arteries are progressively ligatured allowing additional length for advancing the pedicle as necessary. Axillary exposure is maximized with an assistant holding the ipsilateral arm opposite the site of the dissection, thereby facilitating pedicle isolation. Dissection proceeds and the thoracodorsal nerve and circumflex scapular branch are sequentially met. We prefer to isolate the pedicle at the axillary junction; this not only maximizes the vessel length and, in the case of pedicled reconstructions, the arc of rotation, but enables us to preserve the circumflex scapular branch which may permit the addition of an osseous component (if the angular branch is either absent or severed) or scapular/parascapular fasciocutaneous component (Fig 3).

As referred to earlier, the various branches of the subscapular vascular system may be preserved and utilized to add serratus muscle, scapular bone, or scapular/parascapular fasciocutaneous elements. The harvesting of the aforementioned flap components may take place in a sequential fashion as the subscapular pedicle dissection proceeds from caudal to cephalad. Once the serratus arterial branch is met, the surgeon may opt to preserve it to include up to 3 or 4 serratus digitations as part of a chimeric flap; in such cases, the serratus branch is dissected under loupe magnification and the secondary vascular branches preserved up to their entrances into their respective muscle slips. The serratus digitations forming the flap may subsequently be released anterior to posterior from their attachments to the thoracic cage with the help of a periosteal elevator. The angular artery, which is the next branch encountered as the dissection proceeds cephalad, may be followed to the inferior angle of the scapula to raise an osseous flap segment. The scapular attachment of the teres major muscle is cut and detached, thereby exposing the bone's lateral edge and associated distal angular artery. The infraspinatus muscle is then incised according to the dimension of underlying scapular bone desired. The osteotomy is performed with an oscillating saw; importantly, the inferior angle of the scapula should be preserved. The circumflex scapular artery branch is particularly useful for adding a distinct fasciocutaneous component in chimeric flaps. The branch is followed into the triangular space bordered by the teres minor above, teres major below, and long head of the triceps laterally. A corresponding scapular or parascapular skin paddle is designed and harvested. In the case of chimeric flap harvest, this fasciocutaneous component may then be passed retrograde through the triangular space to join the other flap component(s) derived from the subscapular vascular system.

**Figure 4:**
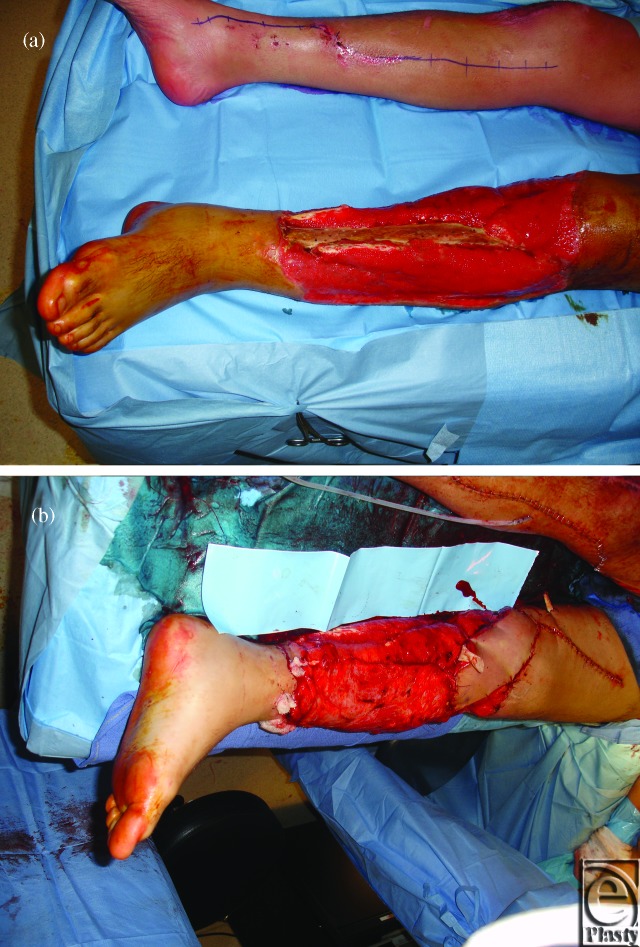
(*a*) Left lower extremity soft-tissue deficit with tibial bone exposure necessitating flap coverage. (*b*) Intraoperative result after chimeric flap inset. The subscapular artery pedicle was anastomosed to the popliteal artery. The contralateral saphenous vein was harvested for additional length in both the arterial and venous pedicles of the chimeric flap.

Finally, once all the necessary flap components have been raised, the pedicle (usually at the level of the subscapular artery) may be divided from its axillary origins with sharp dissecting scissors prior to transposition, if applicable. The thoracodorsal nerve is preserved up to the division of the flap so as to minimize the traction transferred to the pedicle. Figure 4 demonstrates the lower extremity soft-tissue deficit of the depicted patient along with the intraoperative result.

**Figure 5:**
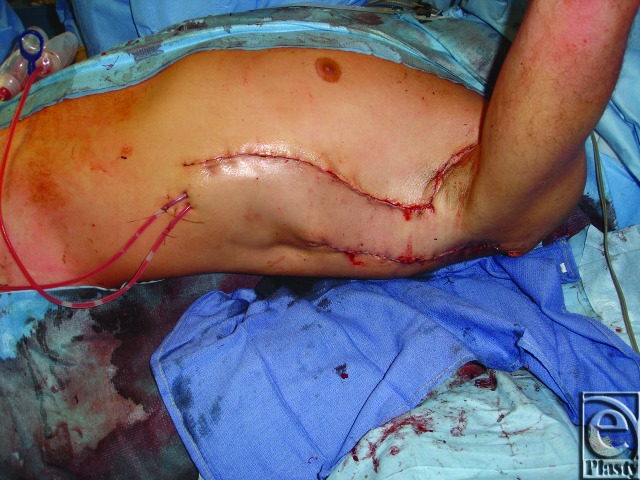
Final donor site closure with 2 Jackson-Pratt drains.

Closure of the donor site is performed following placement of closed suction drains into superficial and deep planes; a third suction drain is placed at the site of raised scapular bone if applicable. A running horizontal mattress suture with 2-0 Vicryl is done on the free anterior margin of residual LD muscle to establish good hemostasis; the muscle edge is then sutured back onto the lateral thoracic wall. Final closure is subsequently completed in 2 planes (Fig 5).

## RESULTS

Over the study period, a total of 105 flaps based on the subscapular vascular system were performed in 105 patients at 2 hospital centers. All flaps were raised using the dorsal decubitus position technique described earlier. Patients consisted of 84 men and 21 women (n = 105), with a mean age of 47.6 years (range: 14-78). Eighty-four flaps were free and 21 were pedicled. Indications for reconstruction included cancer resection, soft tissue loss secondary to trauma, postinfectious deficits, soft tissue loss secondary to burn, upper extremity functional loss, facial paralysis, and recurrent fistula (Table 1). The composition of the harvested flaps included 31 muscular (LD), 49 musculocutaneous (LD), 1 chimeric muscular (LD-serratus), 7 chimeric osteomuscular (scapula-LD), 14 chimeric osteomusculocutaneous (scapula-LD), 2 chimeric musculofasciocutaneous (LD-parascapular), and 1 fasciocutaneous (parascapular). Table 2 summarizes flap tissue composition according to the different anatomical sites reconstructed. The flaps comprising cutaneous components had paddles with a mean surface area of 132.5 cm^2^ (range: 21-460 cm^2^). Multiple skin paddles were harvested in 29 patients. In cases involving a scapular bone segment, mean osseous flap length was 9.8 cm (range: 5-14 cm). Overall, 24 of the flaps in this series were chimeric and contained at least 2 separate tissue components based on their respective arterial pedicles which were all derived from the subscapular system. A 2-team approach was employed in 70 cases (66.7%) with cancer resection or preparation of the recipient site being performed concurrently with flap harvest (Table 3). Intraoperative conversion to the lateral decubitus position was never required in any of the cases.

Overall, 1.9% of patients had minor complications and 8.6% had major complications giving a total rate of 10.5%. Minor complications were restricted to the donor site and included 1 seroma and 1 hematoma. There were 9 cases of arterial (n = 5) and venous (n = 4) thrombosis or insufficiency. Six of these flaps presented with some degree of necrosis; 4 of these were partially salvageable and exhibited only partial flap loss, while 2 of the cases resulted in complete flap loss for an overall failure rate of 1.9% (Table 4).

## DISCUSSION

Over the last 100 years, myocutaneous LDFs and other derivatives of the subscapular system have had a growing range of applications. The significant quantities of available tissue as well as their robust vascular pedicles make these flaps highly useful tools in a wide variety of reconstructive scenarios. However, through innovations in microsurgical technique and evolving 3-dimensional anatomic descriptions of the body's vascular territories, many reconstructive surgeons have moved away from subscapular-based flaps in favor of free tissue transfer from alternate donor sites.

One explanation for the decreasing interest in flaps from the subscapular region is the need to place the patient in the lateral decubitus position to facilitate harvesting. Lee and Mun^15^ attribute this positioning issue as an important factor dissuading microsurgeons from utilizing the TAP flap, thereby partially explaining its infrequent use compared to flaps derived from alternate sites, such as the anterolateral thigh. Several other authors have noted this same drawback in their publications.^4^^,^^16^ Intraoperative position changes in already complex surgeries, such as immediate head and neck reconstructions, present logistical challenges and may increase the level of difficulty as well as the risk of iatrogenic contamination of the operative field. Similar shortcomings of lateral decubitus positioning may be seen during breast reconstruction cases where intraoperative repositioning is required for symmetrization procedures on the contralateral breast.

In our study, we present our experience using the dorsal decubitus position for subscapular-based flap harvest in complex reconstructive cases. To the best of our knowledge, our team is the first to publish and describe this novel solution to the current logistical limitations of subscapular-based flaps. The positioning for this technique is extremely simple, requiring only the installation of 1 or 2 cushions along with full prepping of the ipsilateral upper extremity to allow intraoperative traction as described earlier. Our ability to harvest cutaneous paddles measuring up to 460 cm^2^ and when indicated, scapular bone in complex chimeric flaps, demonstrates the effectiveness of this technique in obtaining sufficient exposure. Using our proposed positioning technique, the surgeon is enabled to harvest the required tissue in an amount equal to that achievable in the traditional lateral decubitus position. Importantly, conversion to the lateral decubitus position was never required in our series; for this reason, we strongly believe that this dorsal decubitus approach is applicable to the full spectrum of subscapular-based flaps. Moreover, the TAP flap can also be raised with the patient placed in this position.

The low total failure rate of 1.9% observed in this study demonstrates the safety of this technique. Overall, 70 combined procedures were performed allowing 2 surgical teams to work simultaneously on separate sites, therefore optimizing operating room time. Since the dorsal decubitus position obviates the need for intraoperative position changes, it may further decrease the procedure length; this benefit also applies to cases that are carried out by a single surgical team. Manipulation of the initial sterile field is also avoided thereby limiting the risk of field contamination. Consequently, patient morbidity may be potentially lessened not only due to decreased time under general anesthesia but by minimizing the chances of postoperative infection. In addition, our technique has possible economic implications as a result of reduced operating room–associated costs. Further studies being planned by our team that prospectively compare harvest times and complication rates between subscapular-based flaps raised in the dorsal decubitus and lateral decubitus positions will serve to shed further light on the proposed benefits of this technique.

## CONCLUSIONS

Flaps based on the subscapular vascular system, whether pedicled or free, are reliable and versatile tools that provide excellent coverage for the reconstruction of a wide range of tissue deficits in various anatomical locations. Harvesting these flaps in the dorsal decubitus position, as described by our team, provides the reconstructive surgeon with an effective and safe method of achieving flaps that match those obtainable in the conventional lateral decubitus position, regardless of the required volume or tissue composition. Furthermore, a 2-team approach is rendered possible in almost any operative scenario. In cases where a subscapular-based flap is deemed the most suitable option for reconstruction, the application of the described dorsal decubitus technique may serve to eliminate many of the limitations associated with the conventional harvesting method.

## Figures and Tables

**Table 1:** Summary of indications for flap reconstruction

Surgical indication	n (%)
Cancer	58 (55.2)
Trauma	32 (30.5)
Infection	9 (8.6)
Burn	2 (1.9)
Functional loss (upper extremity)	2 (1.9)
Facial paralysis	1 (1.0)
Fistula	1 (1.0)
Total	105 (100)

**Table 2:** Tissue composition of flaps, by recipient site

	M	MC	CM	COM	COMC	CMFC	FC	Total flaps (%)
Head and neck	5	21	…	3	14	…	…	43 (41.0)
Axilla	…	4	…	…	…	…	…	4 (3.8)
Breast	…	7	…	…	…	…	…	7 (6.7)
Thorax	…	7	…	…	…	…	…	7 (6.7)
Abdomen	…	6	…	…	…	…	…	6 (5.7)
Upper extremity	1	1	…	1	…	1	…	4 (3.8)
Lower extremity	25	2	1	3	…	1	1	33 (31.4)
Perineum	…	1	…	…	…	…	…	1 (1.0)
All recipient sites	31	49	1	7	14	2	1	105 (100)

CM indicates chimeric muscular; CMFC, chimeric musculofasciocutaneous; COM, chimeric osteomuscular; COMC, chimeric osteomusculocutaneous; FC, fasciocutaneous; M, muscular; MC, musculocutaneous.

**Table 3:** Number of cases executed with 2-team approach, by recipient site

Recipient site	Two-team approach (%)
Head and neck	32 (74.4)
Axilla	1 (25.0)
Breast	1 (14.3)
Thorax	2 (28.6)
Abdomen	2 (33.3)
Upper extremity	…
Lower extremity	32 (97.0)
Perineum	…
All	70 (66.7)

**Table 4:** Summary of complications

Complication	n (%)
**Minor complications**	2 (1.9)
Seroma	1 (1.0)
Hematoma	1 (1.0)
**Major complications**	9 (8.6)
Arterial or venous thrombosis/insufficiency	9 (8.6)
Partial flap necrosis	4 (3.8)
Complete flap necrosis	2 (1.9)
**Total complications**	11 (10.5)
